# Bioactive Sugarcane Lipids in a Circular Economy Context

**DOI:** 10.3390/foods10051125

**Published:** 2021-05-19

**Authors:** Francisca S. Teixeira, Susana S. M. P. Vidigal, Lígia L. Pimentel, Paula T. Costa, Manuela E. Pintado, Luís M. Rodríguez-Alcalá

**Affiliations:** Escola Superior de Biotecnologia, CBQF—Centro de Biotecnologia e Química Fina—Laboratório Associado, Universidade Católica Portuguesa, Rua Diogo Botelho 1327, 4169-005 Porto, Portugal; fsteixeira@porto.ucp.pt (F.S.T.); ptcosta@porto.ucp.pt (P.T.C.); mpintado@porto.ucp.pt (M.E.P.); lalcala@porto.ucp.pt (L.M.R.-A.)

**Keywords:** sugarcane, bioactive lipids, circular economy, terpenes, fatty alcohols, phytosterols, tocopherols, anti-hypercholesterolemic, antioxidant, anti-inflammatory

## Abstract

Most of the global sugar and ethanol supply trade comes from the harvesting of *Saccharum officinarum* (i.e., sugarcane). Its industrial processing results in numerous by-products and waste streams, such as tops, straw, filter cake, molasses and bagasse. The recovery of lipids (i.e., octacosanol, phytosterols, long-chain aldehydes and triterpenoids) from these residues is an excellent starting point for the development of new products for various application fields, such as health and well-being, representing an important feature of the circular economy. By selecting green scalable extraction procedures, industry can reduce its environmental impact. Refluxed ethanol extraction methods have been demonstrated to meet these characteristics. On the other hand, effective non-solvent methodologies such as molecular distillation and supercritical CO_2_ extraction can fractionate lipids based on high temperature and pressure application with similar yields. Sugarcane lipophilic extracts are usually analyzed through gas chromatography (GC) and liquid chromatography (LC) techniques. In many cases, the identification of such compounds involves the development of high-temperature GC–MS/FID techniques. On the other hand, for the identification and quantification of thermolabile lipids, LC–MS techniques are suitable for the separation and identification of major lipid classes. Generically, its composition includes terpenes, phytosterols, tocopherol, free fatty acids, fatty alcohols, wax esters, triglycerides, diglycerides and monoglycerides. These compounds are already known for their interesting application in various fields such as pharma and cosmetics due to their anti-hypercholesterolemic, anti-hyperglycemic, antioxidant and anti-inflammatory properties.

## 1. Introduction

Nowadays, industrial processes that are not aligned with suitable sustainable standards can lead to undesirable consequences such as the pollution of all ecosystems [1] and the release of CO_2_ and other greenhouse gases that are responsible for global warming. Concerns regarding this matter are being raised worldwide in several areas, encouraging the adoption of practices based on renewable concepts [2]. In fact, the current economic paradigm is based on a “take, make, dispose” policy [3] that increases social (i.e., poor working conditions, unemployment, poverty), environmental (water and land exhaustion and pollution) and economic instabilities (supply and financial risks) as a result of the lack of sustainability [4,5]. Thus, in order to assist in the environmental, energy and food production crisis that humankind is facing during the XX and XXI centuries, a new concept has been proposed and developed—the circular economy [6,7].

Geissdoerfer et al. [4] define the circular economy as “a regenerative system in which resource input and waste, emission, and energy leakage are minimized by slowing, closing, and narrowing material and energy loops. This can be achieved through long-lasting design, maintenance, repair, reuse, remanufacturing, refurbishing, and recycling”.

In terms of the protection of natural resources, the abovementioned ideas, actions and strategies seek to result in processes that are more efficient and environmentally friendly. These concepts agree with the principles of green chemistry. Herein, by avoiding the use of toxic solvents, minimizing the number of production steps and designing safer processes, biodegradable residues can be produced [8]. Moreover, the European Union has introduced the circular economy as a high-level strategy to move our societies beyond the limits of growth. The circular economy aims at transforming waste into resources and bridging the activities of production with consumption [9,10].

Within the various industrial activities, we can highlight the agro-industrial sector, where sugarcane residues can be harnessed in diverse areas. Therefore, it is crucial to ensure that this sector abides by the principles of the circular economy [11]. This current work aims to review the state of the art regarding sugarcane production’s impact on global trade as well as its application in the circular economy. Hence, the lipids extracted from this plant are an excellent starting point for the creation of new products, enhancing the economic system for various fields such as health and well-being, as well as new biomaterials.

### 1.1. Sugarcane Production, Residues and Circular Economy

The demand for sugarcane (*Saccharum officinarum* L.)-derived products is increasing worldwide; its production increased from 1.7 to 1.9 billion tonnes from 2008 to 2018. According to statistics provided by the Food and Agriculture Organization (FAO), the main producing countries are Brazil (731 million tonnes) and India (337 million tonnes) [12]. Currently, sugarcane cultivation aims to cover 80% of total sugar supply [13] but its crop and processing results in several by-products and waste streams [14].

**Figure 1:**
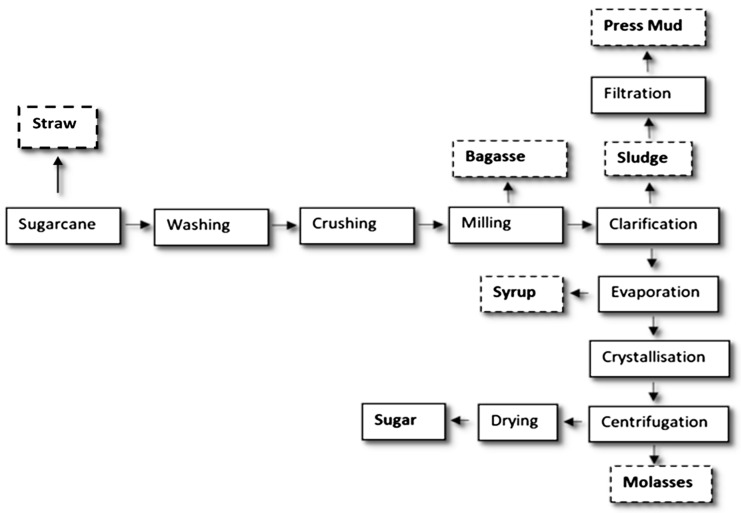
Sugar production process and waste generation [15].

The resulting by-products from harvesting include tops and straw, while the wastes from the sugar processing steps are bagasse, press mud and molasses (Figure 1) [15]. Based on their composition (Table 1), this biomass has potential for use as bio-based products among different sectors, such as food, bioenergy and bio bulk materials [11].

For example, bagasse wastes, which are mainly composed of cellulose (47–52%), hemicellulose (25–28%) and lignin (20–21%) [13], turn bagasse into an interesting raw material because of its low cost and good accessibility related to the constant and stable supply [16]. Furthermore, pre-hydrolyzed bagasse has been reported to be suitable for cattle feeding as it does not interfere with the activity of cellulolytic bacteria of rumen and therefore does not compromise feed digestibility [17].

Press mud, or filter cake, results from slurry that is sent to filtration for sugar removal. It can be used for cane wax production or as a fertilizer in sugarcane fields due to its abundance in nitrogen, phosphorus, calcium and organic matter [15,17].

Sugarcane tops, straw, molasses and bagasse are important materials applied for bioethanol production.

**Table 1:** Sugarcane by-products, lipophilic extract yield (%) and their main bioactive lipids (%).

Sugarcane byproducts	Isolation Method	Extract Yield (%) ^a^	Bioactive Lipids	Biological Effect	Reference
Rind	Supercritic CO_2_	0.80 %	Long-chain aldehydes and n-policosanols (83%)	Prevention of osteoporosis, cardiovascular diseases such as deficient arterial function and hypercholesterolemia [18,19].	[18]
Leaves		1.60 %	Triterpenoids (16.9 %)	Analgesia and anti-inflammatory potential, anti-cancer anti-bacterial activity. Vascularizing agent [18,20].	
Bagasse	Soxhlet (Acetone)	0.90 %	Aldehydes (48%) and n-fatty alcohols (23%)	Fatty alcohols can reduce platelet aggregation, LDL in blood and cholesterol synthesis and prevention of atherosclerosis. Aldehydes are intermediates in the biosynthesis of alcohols from fatty acids [21]. Phytosterols act as anti-inflammatory agents on macrophages (increase of phosphatase SHP-1 activity, secretion of anti-inflammatory interleukin IL-10, reducing transcription factor activation and decrease on the release of pro-inflammatory cytokines IL-12 and IL-5) [22,23].	[21]
Straw		1.40 %	Fatty acids (60%), sterols (10%)		
Peel	Saponification and further extraction with Diethyl Ether	0.027 %	Octacosanol (81%)	Increment of HDL levels and decrease of LDL and triglycerides. Reduction of oxidative stress by the increase on superoxide dismutase enzyme levels [18,19].	[24]
Filter Mud	Saponification and further extraction with Ethanol	2.31 %	Octacosanol (47.8 %)		[25]

^a^ of the dry biomass weigh.

### 1.2. Ethanol Production

Environmental and fossil fuel supply issues have led to an increase in global bioethanol production since 2014 from 789.9 to 916.4 tonnes in 2019, from which 54% came from US, 30% from Brazil and only 5% from the EU [26]. The evolution of the biofuel sector over the past decade has been strongly influenced by various policies, including supporting legislation measures and mandatory blending levels. Moreover, during the past decade, the use of ethanol as an octane additive has increased rapidly, due to the high fossil fuel prices [27]. Consequently, it is expected that there will be an increase in the worldwide consumption of ethanol from 111.5 to 129.2 million liters, of which 102.3 million liters will be used as biofuel [28].

Bioethanol production from sugarcane fermentation is a paradigm in the implementation of a strategy to bypass the depletion of fossil fuels, in order to obtain replacement oils for spark-igniting engines (i.e., gasoline) [29]. The first-generation biofuels are obtained from seeds, grains or sugars, which are used for foodstuff consumption, leading to some concerns about biodiversity and land usage [30,31]. Currently, first-generation biofuels are mostly produced from sugar and starch-containing raw materials such as molasses that result from sugar refining, and they have been recently reported as suitable to for generating bioethanol by fermentation processes [32,33]. On the other hand, the second-generation biofuels make use of biomass and have recently attracted much interest, since there is no need for extra land or competition with food/feedstock. An example is the bioethanol production from lignocellulosic biomass (i.e., bagasse, straw) [34]. Further, both bagasse and straw are basically constituted of lignin, hemicellulose and cellulose, being suitable for producing second-generation biofuels through hydrolysis processes. Here, hemicellulose and cellulose are converted into sugars to afterwards be transformed into ethanol by fermentation processes [34].

Some studies reported that processes using sugarcane and bagasse (second-generation) present similar ethanol yields as the ones using sugarcane molasses (first-generation) [35]. However, while cellulose hydrolysis produces glucose, which can be easily fermented into ethanol, hemicellulose hydrolysis produces mostly xylose. Unfortunately, only a few microorganisms are capable of transforming xylose into ethanol [17], and additional research will be needed concerning the tolerance of such microbial strains to substrate-hydrolysates to further improve the second-generation biofuel production [29]. Despite the past four decades of work and significant methodology development, large industrial-scale processes for fermenting pentose and hexose still face technical and economic challenges, predominantly aiming at the improvement of biomass pre-treatments [36].

Moreover, sugarcane lipids have been recently demonstrated to be suitable in the framework of the bioeconomy. On the basis that it is a high-biomass crop, this plant may exceed oil production from other oilseed harvests (i.e., soya), representing a way to decrease biofuel production costs [37]. Sugarcane oil accumulates in leaves and stems and is composed of phospholipids (that can be eliminated with acetone during biofuel production), triglycerides and free fatty acids, the latter being further converted into diglycerides [38]. Moreover, other initiatives based on genetic engineering optimized lipid production to reach up to 20% [39]. This is a remarkable improvement since regular content increases from 0.1% to 0.3% depending on the variety, although the composition is far more complex since it includes fatty alcohols, phytosterols, ketosteroids, hydroxyketosteroids and terpenoids [40]. Biodiesel is obtained through reactions where fatty acids in the triglycerides are transesterified into fatty acid methyl esters in the presence of an alcohol (usually methanol in 6:1 ratio) and a catalyst, with sodium methoxide (0.5%) being the most common [41]. Interestingly, in the first days after the creation of the diesel motor, vegetable oils were proposed as possible fuels but their viscosity was a major problem (i.e., operational problems due to deposits) and the elimination of glycerol after transesterification reactions was then proposed [41].

Oils from soya and rapeseed are widely used to produce biodiesel, with net energy outputs of 2.6 and 6.6. GJ/tonne crop, while with bioethanol from sugarcane, values are 2.3 GJ/tonne crop [42]. However, in terms of harvested land sugarcane yields, this is 153 GJ/ha, while for soya oil intended for biodiesel, this yield is 6.5 GJ/ha, and it is 15.3 GJ/ha for rapeseed. Thus, as sugarcane is a high-yield crop (as commented above), the studies where lipid production was boosted represent an important development in energy production from vegetable sources.

However, both biodiesel and bioethanol production require a great deal of energy due the fermentative or chemical steps (among others). Thus, the carbon footprint of bioethanol from sugarcane is 14 kg CO_2_eq/GJ, and in the case of biodiesel, it is 130 kg CO_2_eq/GJ (i.e., soya) and 87 kg CO_2_eq/GJ (i.e., rapeseed). It is therefore expectable that if sugarcane could be used to produce oil suitable for biodiesel, the carbon footprint would be lower than with the current harvested plants [42]. Moreover, other environmental indicators such as the blue (i.e., surface and groundwater consumed during production), green (i.e., rainwater consumed during production) and grey (i.e., freshwater pollution due to production) water footprint are favorable to sugarcane. Indeed, the total water footprint in sugar crops during the period 1996 to 2005 was 193 m^3^/tonne and 2364 m^3^/tonne for oil crops [43].

Accordingly, sugarcane lipids, simply through their direct application in energy production, can make a great impact within the bioeconomy since current information predicts lower land, water and carbon footprints. Nevertheless, it can be also expected that if genetic engineering brings new varieties of sugarcane with higher lipid yield, its bioproducts will also have higher contents. Until that moment arrives, exploring current possibilities can help to set in motion such opportunities.

## 2. Sugarcane Lipophilic Molecules

**Figure 2:**
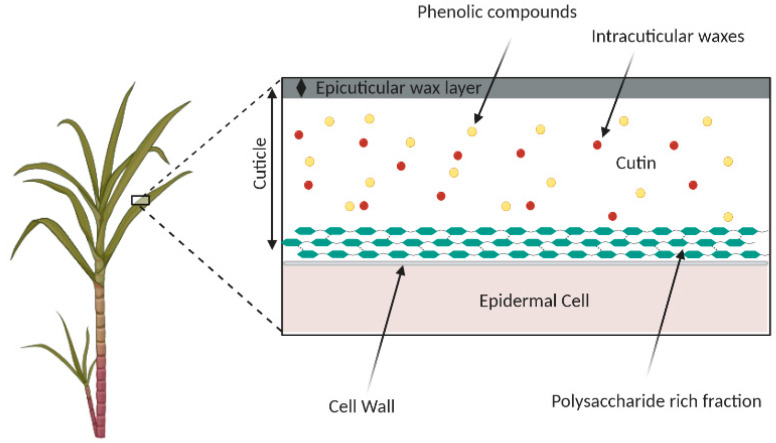
Representation of the cross-section of sugarcane plant cuticle [48].

Lipids and their applications have recently attracted significant interest in several research fields since their diverse biological pathways in cell biology, physiology and pathology are being revealed [44]. A wide-ranging variety of lipids can be present in plants; these include hydrocarbons, wax esters, fatty alcohols, ketones, free sterols and sterol esters, as well as more common simple lipids such as triglycerides. Among these, wax esters are the main components of the surface lipids of plants [45], covering the epidermal cells of fruits, petals and leaves, designing the cuticle matrix. The cuticle (Figure 2) acts as a physical barrier and signaling trigger against pathogens (i.e., bacteria, yeast, fungi, virus and mites) and environmental conditions (i.e., wind and rain) [46,47]. It consists of an amorphous and non-soluble polyester formed by polyhydroxylated fatty acids C16 and C18—denominated as the cutin matrix [48,49]. The biosynthetic pathways from fatty acids to cutin monomers are divided according to three reactions: coenzyme-A (CoA) conjugation, oxidation and acyl transfer to glycerol [50]. The connection between polymer structures, functions and properties is a promising area of research [51].

### 2.1. Classic Methods for Lipid Isolation

**Figure 3:**
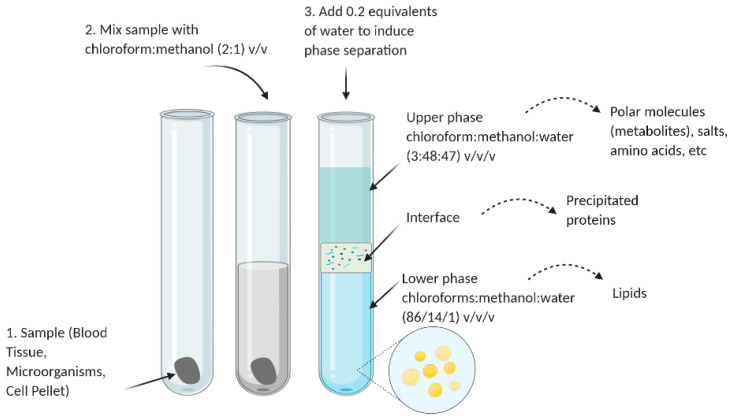
Representation of Folch’s extraction method [55].

In the isolation of lipids, Folch extraction (Figure 3) is the gold standard [52]. The procedure, originally developed to be assayed on biological samples, takes advantage of a biphasic solvent system consisting of chloroform:methanol:water [53]. Based on this idea, a rapid and less solvent-consuming method was proposed in 1959 by Bligh and Dyer [53,54]. This method was performed on wet tissue homogenized with a mixture of chloroform and methanol, forming a miscible system of chloroform:methanol:water at a 2:2:1.8 (*v*/*v*/*v*) proportion. 

Later, in 1978, in order to review these techniques as potential health hazards due to the use of toxic solvents, Hara and Radin [55] proposed an improved extraction method using hexane:isopropanol 3:2 (*v*/*v*).

More recently, a modification to the Folch procedure using methyl tert-butyl ether instead of chloroform was also proposed—the Matyash method [56]. Due to its lower density compared to the methanol/water phase, it was possible to recover the lipid layer in an easier way. Later on, a modified Matyash method, using solvent volumes analogous to the Bligh and Dyer protocol, was studied in order to achieve better reproducibility for metabolite analysis by UPLC–MS on lipid extracts of two bio-fluids (human plasma and urine) and on Daphinia magna tissue [57]. The improved reproducibility was evaluated based on the observation of a lower median relative standard deviation (mRSD) of peaks identified by UPLC–MS.

Nonetheless, there are two major challenges to overcome: (1) the extraction efficiency and (2) the complete removal of non-lipid content. For example, phospholipids can bind to various biopolymers through ionic interactions that cannot be easily disrupted by organic solvents. In this context, pH adjustments during extraction can be beneficial for lipid maximal recovery and can also increase the ionization efficiency of lipids in mass spectrometry analysis [58]. For instance, the acidified Bligh and Dyer procedure can be used in order to extract the lipid content of Saccharomyces yeast cell homogenates, entailing the acidification of the aqueous phase with HCl (0.1 M) to ensure efficient extraction of the acidic phospholipids [59].

Regarding these solvent-assisted extractions, Reis et al. [60] evaluated five solvent systems for the extractability of lipids accomplished with further analysis by HPLC–MS. A comparison between the Folch, Bligh and Dyer, acidified Bligh and Dyer, Matyash and Hara and Radin methods showed that there was a similarity in the predominant lipid classes (triglycerides, cholesterol esters and phosphatidylcholines) but differences in the extraction of minor lipid classes (phosphatidylinositols, lysolipids, ceramides and cholesterol sulfates). Both the Folch and acidified Bligh and Dyer methods had the best extraction efficiency over the range of lipid classes.

Other extraction techniques using isopropanol, acetone, ethanol, methanol and acetonitrile have been identified for their lipophilic selectivity depending on the solvent proportions, applied extraction temperatures and sample preparation [61,62].

One of the major disadvantages is that these methods are, in general, intended as lab-scale methods for analyses of the lipid extracts and not as procedures to be used at large scale. Moreover, these isolation methods use organic solvents that are either derived from petroleum or toxic and do not fit into an environmentally friendly strategy.

**Figure 4:**
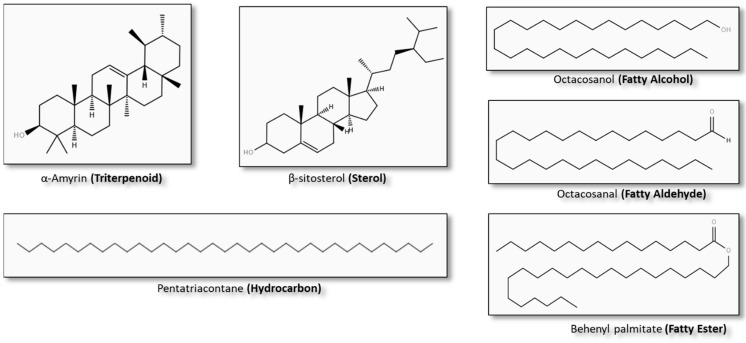
Examples of chemical compounds from plant waxes. Structures: (The LIPID MAPS Lipidomics Gateway, 2020).

In the following sections, the most common methods to isolate sugarcane lipids (Figure 4) from by-products will be discussed, with special attention to those that can be used at industrial scale and fall into a green chemistry strategy.

### 2.2. Environmentally Friendly Extraction Methods

Innovative green procedures can be scaled in order to produce major quantities of the final product and have been developed to reduce time, solvent and energy production consumption [63].

In this context, Lake et al. [64] proposed a process to recover wax from the sugarcane filter cake involving a heated aqueous solution with surface active agents such as kerosene.

Soxhlet is one of the conventional techniques for extracting products from biomass. A cellulose thimble is loaded with the plant material and placed in a Soxhlet extractor, which is directly connected to a solvent reservoir. The solvent is heated to its boiling point and condenses successively, which improves extraction and leads to lower solvent consumption [65]. The procedure can be carried out in repeated cycles with different solvents in order to improve the purification of the crude wax (i.e., removing chlorophyll) [25] and/or selectively obtain a sample fractionation [66].

Nuissier et al. [66] obtained a crude wax from sugarcane rum factory wastes by Soxhlet using cyclohexane for 20 h with 7% wt. yield. Within the Soxhlet technique, a mixture of hexane:methanol (20:1 *v*/*v*) was used for 16 h by Asikin et al. [67] in order to extract the maximum amount of wax and consequently policosanol and long-chain aldehyde of the rinds of several sugarcane cultivars.

The temperature of the procedure can also influence the extract yield. To prove this, Holser and Akin [68] studied the effect of the extraction temperature on the total lipid content, concluding that the total amount of lipids extracted usually increased with the extraction temperature. The extraction was carried out using a less toxic solvent, ethanol, where, accordingly, extractions from flax cuticle at 50, 80 and 90 °C yielded 7.6, 14.7 and 19.4 mg lipids/g solids, respectively.

Octacosanol extraction from filter mud can be performed by refluxed ethanol (5.35 ± 0.06 g/100 g of wax yield) followed by wax purification and saponification [25]. Likewise, a patent published in 1999 describes the isolation of primary aliphatic alcohols by saponification and further extraction with organic solvents from sugarcane wax [69].

Supercritical carbon dioxide extraction has been proven as a potential method to extract compounds of interest from herbs, such as vitamin E from Pithecellobium Jiringan extracts [70]. Attard et al. [65] found that, for the majority of compounds, supercritical CO_2_ extracted larger amounts of lipophilic molecules than the Soxhlet method using hexane as a solvent. Moreover, applying supercritical extraction on sugarcane leaves, the wax yield was 1.60% of the dry biomass. Moreover, another study concluded that ethanol reflux extraction and supercritical CO_2_ extraction can provide analogous wax yields [25]. In order to increase the yield of supercritical CO_2_ extraction, a co-solvent can be added. Thus, a commercial-scale sugarcane wax extraction from the mud of sugar mill presses was achieved using ethanol as a co-solvent [71].

A technique involving immersion cycles in liquid nitrogen and extraction with n-hexane permitted wax removal from the plant cuticle. The wax yield significantly increased with the increase in the number of immersion cycles, with the highest wax yield being achieved after six immersion cycles in liquid nitrogen: 26 ± 1 and 19 ± 3 mg of wax per 100 g of wheat and flax straw, respectively. In this case, crystallization (principally of β-diketones) and wax-cracking by liquid nitrogen immersion cycles may have played an important role in epicuticular wax removal [72].

Solid-phase extraction, microwave-assisted extraction and other technologies are used to extract lipids from samples [73]; nevertheless, it is crucial to perceive that wax yields and its composition may fluctuate depending on the cultivar, the specific morphology of the part of the sugarcane analyzed and the degree of plant maturity [18,67].

### 2.3. Characterization of Lipophilic Sugarcane Extracts

The characterization of the lipophilic extracts can be attained using lipidomic tools. Lipidomics is often discussed separately because lipids require specific sample preparation, analytical protocol and data processing [74]. In order to expand the insight of the numerous existing lipid classes (wide variety of size and polarity), its analysis becomes crucial; usually, it requires a combination of different techniques [45]. For reliable lipid analysis, previous fractionation steps are commonly required in order to focus on specific molecules or to avoid ionic suppression. Thus, thin-layer chromatography (TLC) and solid-phase extraction (SPE) are widely used; however, they are considered time- and sample-consuming, involving specific materials [62]. Regarding chromatography techniques, a variety of column-packing materials, eluents and detectors have been used for lipid class separation and further analysis by high-performance liquid chromatography (HPLC) and/or gas chromatography (GC) [45]. Table 2 summarizes some characterization methods employed according to the target analyte, namely triterpenoids, phytosterols, tocopherol, glycolipids, steryl glucosides and glucosyl-ceramides as well as alkane, fatty acids, esters, aldehydes and alcohols.

Río et al. [21] studied the lipophilic phytochemicals from sugarcane straw and bagasse wastes. Bagasse lipophilic extracts were mainly composed of n- aldehydes (48% of all identified lipids) and n-fatty alcohols (23%), steroid ketones (14%) and n-fatty acids (10%), while straw extracts contained n-fatty acids (60% of all identified compounds), sterols (10%) and steroid ketones (14%). Other researchers reported the presence of policosanol, a mixture of high-molecular-weight aliphatic alcohols (e.g., octacosanol and triacontanol) in sugar cane peel in quantities of 270 ± 4 mg/kg and in sugar cane leaves of 181 ± 4 mg/kg [24]. Policosanol has been used in nano-emulsions from rice bran wax for the controlled delivery of pharmaceutical and cosmetic ingredients, as reported by Ishaka et al. [75], leading to a new pathway for the policosanol bioactivity of sugarcane wax.

Pensec et al. [76] identified and quantified triterpenoids by GC–MS/FID of leaf cuticular waxes from Vitis vinifera cultivars. After extraction with chloroform, the extracts were separated by preparative thin-layer chromatography on silica gel into three fractions: (1) free steroids and neutral triterpenes, (2) triterpene acids and (3) triterpenoid low-polarity esters. These samples were injected at a ratio of 1:10 into an HP-5MS column with helium as a carrier gas at a flow rate of 1 mL/min. The fraction containing free steroids and neutral triterpenes was characterized by the presence of high levels of lupeol and taraxerol.

Phytosterols such as stigmasterol, sitosterol, 3,6-diketosteroids, Δ4-3-keto steroids and Δ4-6-hydroxy-3-keto steroids have also been identified in sugarcane wax from filter cake press mud [77].

Attard et al. [18] analyzed sugarcane waxes by high-temperature GC–MS quadrupole mass spectra operated in the electron ionization mode (EI), identifying fatty acids, alcohols, alkanes, aldehydes, wax esters, sterols and triterpenoids. This analysis showed that crusgallin was the most abundant triterpenoid present in lipophilic leaf extracts. Regarding saturated fatty acids and sterols, hexadecanoic acid and β-sitosterol were the most abundant compounds, respectively.

Nuissier et al. [66] isolated three fractions of crude wax from sugarcane distillation wastes, concluding that solvents of different polarity led to different wax compositions. The composition of the fraction soluble in methanol consisted of palmitic fatty acid esters, pentacyclic triterpenoids (cylindrin and crusgallin), sterols (β-sitosterol, stigmasterol and campesterol) and free fatty acids. On the other hand, the fractions obtained with acetone contained C20-C29 alkanes and phytosterols. These results were obtained by GC–MS/FID on a CP Sil. 5CB column (25 m × 0.25 mm inner diameter, 0.25 µm film thickness) at oven temperatures from 150 to 320 °C using helium as a carrier gas.

Another study, performed by Asikin et al. [67], examined the sugarcane wax composition on policosanol and long-chain aldehydes by GC–MS/FID on a fused capillary column DB-5 (30 m × 0.25 mm inner diameter, 0.25 µm film thickness) with temperatures from 150 to 320 °C using helium as a carrier gas. The total policosanol contents of sugarcane rind samples ranged from 60 to 124 mg/100 g, with octacosanol being the main alcohol, while total aldehydes ranged from 70 to 115 mg/100 g, with octacosanal being the main aldehyde present.

**Table 2:** Lipophilic plant analysis characterization methods.

Analyte	Matrix	Method	Column	Carrier Gas/Mobile Phase	Detector	Reference
Triterpenoids	Leaf cuticular waxes of Vitis vinifera cultivars	GC–MS/FID HPLC	HP-5MS (30 m × 0.25 mm i.d., film thickness 0.25 μm) Synergy MAX-RP 80A Phenomenex (250 mm × 4.6 mm i.d., 4 μm)	He Acetonitrile	FID, MS UV–Vis (200, 254 nm)	[76]
	*Hieracium pilosella* L.	GC–MS/FID	RTX-1 (30 m × 0.32 mm i.d., 0.25 μm)	N_2_	FID, MS	[78]
	Tamus edulis	GC–MS/FID	HP-5MS 30 m × 0.25 mm i.d., 0.25 μm)	H_2_	FID, MS	[79]
Phytosterols, tocopherols	Mango	GC–QTOF–MS	RTX-200MS (30 m × 0.25 mm i.d., 0.25 μm) HP-5MS (30 m × 0.25 mm i.d., 0.25 μm)	He	QTOF	[80]
	*Saccharum officinarum* L.	GC–MS	Zebron (ZB5 MS) (30 m × 0.25 mm i.d., 0.25 μm)	He	MS	[77]
Glycerolipids, steryl glucosides and glucosyl-ceramides	Arabidopsis thaliana	LC–IT–TOF–MS	HILIC (100 mm × 2.1 mm i.d., 3 µm)	A—Methanol:water (95:5, *v*/*v*) B—Acetonitrile:methanol:water (95:2:3, *v*/*v*/*v*)	MS IT–TOF	[81]
Alkanes, fatty acids, fatty alcohols, ester, aldehyde and alcohol	*Saccharum officinarum* L.	GC–MS	RTX-5MS (30 m × 0.25 mm i.d., 0.1 µm)	He	MS	[82]
		GC–MS	DB5HT (30 m × 0.25 mm i.d., 0.25 μm)	He	M	[18]
		GC–FID GC–MS	DB5HT (5 m × 0.25 mm i.d., 0.1 μm) DB5HT (15 m × 0.25 mm i.d., 0.1 μm)	He	FID MS IT–TOF	[21]
		GC–MS	Equity-5 (30 m × 0.25 mm i.d. × 0.5 μm)	He	MS	[24]
		GC–MS/FID	CP Sil 5 CB (25 m × 0.25 mm i.d., 0.25 μm)	He	FID, MS	[66]
		HPLC GC–MS/FID	Luna (250 mm × 4.6 mm i.d., 5 μm) DB-5 (30 m, 0.25 mm i.d., film thickness 0.25 µm),	A—Hexane B—Methyl tert-butyl ether containing 0.2% acetic acid. He	ELSD FID, MS	[67]
	Soybean	HPLC	Inertsil Si. (150 mm × 2.1 mm i.d., 5 μm)	A—Isooctane:ethyl acetate (99.8:0.2, *v*/*v*) B—Acetone:ethyl acetate (2:1, *v*/*v*) C—Isopropanol:water (85:15, *v*/*v*) D—Ethyl acetate	ELSD and Corona CAD LTQ-Orbitrap with ESI, APCI/APPI ion source	[83]
	Rice bran	GC–MS	Equity-5 (30 m × 0.25 mm i.d. × 0.5 μm)	He	GC–MS	[75,84]

As mentioned previously, lipids are complex mixtures of individual classes; thus, some cannot volatilize using gas chromatography. For this reason, liquid chromatography coupled to mass spectroscopy (LC–MS) has become important for lipid analysis [45]. Okazaki et al. [45] performed LC–MS–IT–TOF analysis with an HILIC column using methanol:water 95:5 (*v*/*v*) and acetonitrile:methanol:water 95:2:3 (*v*/*v*/*v*) as mobile phases. This method allowed the identification of monogalactosyldiacylglycerol, steryl glucoside, glucosylceramide, phosphatidylglycerol, digalactosyldiacylglycerol, phosphatidylethanolamine, phosphatidylinositol and phosphatidylcholine in the lipophilic extract of A. thaliana roots and leaves. These results indicated that HILIC–MS analysis was suitable for the investigation of the plant lipidome. Since there is a wide polarity range in lipid classes, to achieve the desired separation in a shortened timeframe, it is necessary to use complex mobile phase mixtures and elution gradients.

The lipid classes from sugarcane wax were also evaluated by HPLC using an Evaporative Light Scattering Detector (ELSD) and a silica column (250 mm × 4.6 mm i.d., 5 µm). The method allowed the identification of lipid classes as follows: 55–60% aldehydes and sterol esters, 32–40% alcohols and lower percentages of triglycerides and free sterols [67].

An HPLC system coupled to a UV–Vis detector was used to achieve quantitative analysis of triterpenoids (α-amyrin and lupeol) using an isocratic system (100% acetonitrile) with a synergy MAX-RP 80A phenomenex column (250 mm × 4.6 mm i.d., 4 µm) [76]. 

In combination with omics analysis, the biophysical approach using Fourier transformed infrared (FTIR) spectroscopy and differential scanning calorimetry (DSC) can be applied to these samples in order to obtain more detailed knowledge of the plant cuticle [85].

FTIR allows the identification of organic functional groups demonstrating a parallelism to the respective composition [82]. Aliphatic chains may be identified by the presence of –CH stretching vibration bands at 2921.73 and 2851.64 cm^−1^ and at 1463.44 and 1376.96 cm^−1^ due to –CH2 and –CH3 bending vibration. Stretching bands at 3395.60 cm^−1^ indicate the presence of alcoholic groups (-OH) in waxes. –C=O stretching vibration bands at 1710.25 and 1736.63 cm^−1^ may represent different functional groups, namely aldehydes, carboxylic acids and esters [82].

With DSC analysis, it is possible to determine the thermal characteristics (melting, crystallization and decomposition temperatures) and the respective enthalpies for each phase transition associated with heat absorption (endothermic) or liberation (exothermic). When applied to waxes, fusion temperatures can give information about the presence of coating agents [86]. In fact, the melting point of a wax is inherent to the variety of compounds present and may predict its structure [87]. Accordingly, Attard et al. [18] identified by DSC a range of melting points on sugarcane wax, varying from 63 to 76 °C.

Parameters such as oxidation induced time (OIt) and oxidation induced temperature (OIT) can be also determined through DSC analysis, using an oxygen atmosphere [88].

## 3. Sugarcane Lipids as Bioactive Agents

The lipophilic compounds of *Saccharum officinarum* L. are described as anti-hypercholesterolemic and anti-hyperglycemic [40]. Design strategies to predict these activities are essential after performing compositional analysis.

In the following section, we present some biological studies to evaluate these functions.

### 3.1. Hypocholesterolemic, Antioxidant and Anti-Hyperglycemic Properties

A comprehensive understanding of the biochemical reactions involved in disordered metabolic pathways, such as cholesterol biosynthesis or glucose uptake, is extremely important for the development of new approaches related to the development of drugs to control hypercholesterolemia and diabetes [89,90].

There has been an increasing prevalence of diabetes mellitus over recent decades, and it has been estimated that the number of patients will increase to 366 million by 2030 [91]. Zheng et al. [92] evaluated the capacity of sugarcane bagasse hydro-alcoholic extracts to access intestinal α-glucosidase inhibitory activity, concluding that it had anti-hyperglycemic capacity.

The pharmaceutical, nutraceutical and cosmetic industries are becoming alert to the applications of triterpenoids as functional molecules in many healthcare products [93]. Their activity has been demonstrated through in vitro and in vivo studies (predominantly on mice models). For example, the antidiabetic activity carried out on a diabetic mice model enhanced higher plasma and pancreatic insulin concentrations, leading to the preservation of pancreatic β-cells [20]. Assessing the insulin sensibility by controlling insulin receptors (IR) may predict the antidiabetic function. Therefore, Jung et al. [94] demonstrated that bioactive extracts from plants, mainly composed of pentacyclic triterpenoids, led to the activation of insulin-mediated tyrosine phosphorylation of the IR β-subunit. Furthermore, ursolic acid behaved as an insulin sensitizer at doses as low as 1 µg/ml in a cellular model of insulin-sensitive adipose tissue.

Lipids, such as policosanol, can also represent promising therapeutic targets for obesity and related metabolic disorders such as diabetes. Research considered these properties by studying high-fat-diet-fed mice treated with octacosanol, observing lower body fat gain, hepatic lipid content and insulin resistance related to the increase in brown tissue activity and the improvement of hepatic lipid metabolism [95]. Additionally, Lee and co-workers conducted a study on the impact of octacosanol supplementation in taekwondo athletes subjected to rapid weight loss through caloric restriction and high-intensity exercise training. This latter research reported that there was an improvement of the lipid profile related to the increase in high-density lipoprotein (HDL) levels and decrease in low-density lipoproteins (LDLs) and triglycerides. A reduction in oxidative stress (an increase in superoxide dismutase) was also observed with octacosanol intake of 40 mg for six days when compared to the placebo control group [19].

The bioactivity of phytosterol lipids has also been widely discussed. Functional foods containing phytosterols, i.e., margarines and dairy foods, gained relevance as a result of clinical studies that confirmed their cholesterol-lowering properties [96]. Hence, a meta-analysis concluded that the fat matrix is a determinant of the hypo-cholesterolemic effects of phytosterols and has to be taken in account [96].

The phytochemistry of sugarcane wax include also flavonoids, flavone-glycosides and phenolic acids [40]. In fact, sugarcane bagasse has a high level of total phenolic and total flavonoid content that contributes to its antioxidant and anti-proliferative properties [97]. For example, tricin, a naturally occurring flavone present in rice and sugarcane, has demonstrated chemopreventive properties against murine gastrointestinal carcinogenesis [98,99].

### 3.2. Anti-Inflammatory Properties

Unravelling methods that are involved with lipidic signaling inflammation pathways is a promising research field that can lead to future therapeutics for various diseases. Molecules synthesized from arachidonic acid (AA; 20:4), classified as eicosanoids, are involved in acute immune responses, pain perception, blood pressure regulation, coagulation and reproduction [100,101]. The endogenous bioactive lipids such as eicosanoids, pro-resolving lipid mediators, lyso-glycerophospholipids/sphingolipids and endocannabinoids have been largely related to cellular and molecular mechanisms connected to the pathogenesis of chronic disorders [102,103].

**Figure 5:**
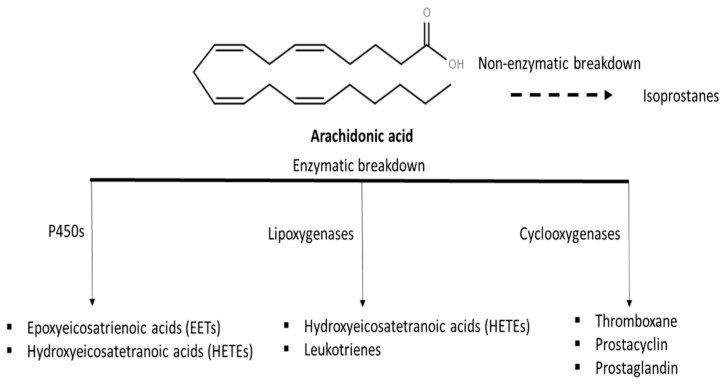
Schematic illustration of transformation of arachidonic acid through non-enzymatic and enzymatic pathways [105]. Structures: (The LIPID MAPS Lipidomics Gateway, 2020).

Eicosanoids result from the enzymatic or non-enzymatic oxidation of very long-chain fatty acids (e.g., arachidonic acid) that can be found in phospholipids of cell membranes as well as being involved in signaling processes [104]. The release of AA from the membrane is driven by the activation of specific receptors that promote the activation of lipolytic enzyme phospholipase A2 (PLA2), which ultimately hydrolyzes the sn-2 ester bond in the phospholipid and releases AA as a free fatty acid. The release of AA leads to the formation of numerous lipidic mediators that trigger inflammation via non-enzymatic (isoprostanes) or enzymatic pathways (epoxyeicosatrienoic acids, hydroxyeicosatetranoic acids, leukotrienes, thromboxanes, prostacyclin and prostaglandin), as schematized in Figure 5 [105,106]. From these mediators, prostaglandins and leukotriene B_4_ are involved in the initial steps of acute inflammation responses that promote changes in blood flow, edema and leukocyte recruitment, specifically neutrophils, at the site of injury. During inflammation resolution, specialized pro-resolving mediators are produced to control the early events in the acute inflammation response, such as edema, leukocyte trafficking and functions related to macrophage phagocytosis, contributing to the homeostasis of the tissue [107]. Macrophage cells are major producers of eicosanoids and other related lipid mediators during inflammation, receiving particular focus in terms of understanding immunity and inflammation because of their central role and dynamic functionality [104].

There is a large potential of anti-inflammatory and immunomodulatory agents within natural sources such as sugarcane plant (*Saccharum officinarum* L.) as well as terpenes derived from the yeast Saccharomyces cerevisiae. Research carried out by Ledon et al. [108] tested the anti-inflammatory effects of bioactive lipids present in the sugarcane plant on in vivo experimental mice with induced arthritis and psoriasis, suggesting a possible correlation between such anti-inflammatory effects and the inhibition of AA metabolism.

The bioactivity of β-sitosterol as an anti-inflammatory agent on macrophages is possibly explained by the increase in phosphatase SHP-1 activity, secretion of anti-inflammatory interleukin IL-10, reducing transcription factor activation and thus the decrease in the release of pro-inflammatory cytokines (IL-12 and IL-5) [22]. Therefore, dietary combined n-3 polyunsaturated fatty acids and plant sterols can influence the reduction of pro-inflammatory markers such as C-reactive protein (CRP), tumor necrosis factor A (TNF-A), interleukin-6 (IL-6) and leukotriene B_4_ (LTB_4_) and the increase in adiponectin, a protein involved in the regulation of glucose levels and fatty acid breakdown, in hyperlipidemic individuals [23].

Some studies have similarly shown that the oral administration of octacosanol (100 mg/kg/day) can inhibit the expression of pro-inflammatory cytokines in the colonic tissues of dextran sulfate sodium-induced colitis mice models. This mechanism of action is perhaps linked to its protective effect on oxidative stress reactions in intestinal cells [109]. Moreover, future research on octacosanol/policosanol should be established along with target markers for inflammation [19].

## 4. Conclusions and Future Perspectives

Currently, *Saccharum officinarum* L. cultivation covers sugar supply and bioethanol production, generating waste streams and by-products that can be introduced in the circular economy paradigm, bridging production and consumption activities while bypassing waste accumulation.

The complex mixture of different compounds in the lipid extracts, as well as their interactions with the containing matrix, have to be specifically adapted to the studied material [61,62,110,111].

Classic extraction methods are, in general, intended as lab-scale methods for further analyses of extracts and not as procedures to be used at a large scale. The most common methods used to isolate lipids from sugarcane by-products are especially those that can be used at industrial scale and that fall into a green chemistry strategy. Refluxed ethanol can be an optimal extraction method in order to achieve better extraction yields and a great variety of compounds.

In accordance with the relevance of lipids, the new research branch of omics science known as lipidomics has proven to be crucial for the identification of novel lipids and for the discovery of potential new sources using proper tools [73].

Multiple column packing materials, mobile phases and detectors have been assayed for lipid class separation by high-performance liquid chromatography (HPLC) and/or gas chromatography (GC) [45]. Previous fractionation steps are commonly used as a way to focus on specific sub-families or avoid ionic suppression. Therefore, thin-layer chromatography (TLC) and solid-phase extraction (SPE) are widely used but time-, sample- and solvent-consuming [62]. 

A wide-ranging diversity of lipids present in this sugarcane matrix include hydrocarbons, wax esters, fatty alcohols, ketones, sterols and sterol esters, as well as the more common, simple lipids such as triglycerides. 

Further characterization of structural and biophysical properties will allow to predict their most suitable applications in a variety of research fields, as well as to unveil their diverse biological pathways in cell biology, physiology and pathology [44]. Additionally, applications in the cosmetic, nutraceutical and pharmaceutical areas are of great interest due to their promising properties as anti-hypercholesterolemic, anti-hyperglycemic and anti-inflammatory agents.

## Abbreviations

AA—arachidonic acid; APCI—atmospheric pressure chemical ionization; CoA—Coenzyme-A; CRP—C-reactive protein; DSC—Differential Scanning Calorimetry; EI—electron impact; ELSD—evaporative light-scattering detector; ESI—electrospray ionization; FAO—Food and Agriculture Organization; FID—flame ionization detector; FTIR—Fourier transform infrared; GC—gas chromatography; HDL—high-density lipoprotein; HILIC—hydrophilic interaction liquid chromatography; HPLC–MS—high-performance liquid chromatography–mass spectrometry; hsCRP—high-sensitivity C-reactive protein; IL—interleukin; IR—insulin receptors; LC–IT—liquid chromatography ion trap; LDL—low-density lipoprotein; LTB_4_—leukotriene B_4_; LTQ—linear trap quadrupole; mRSD—relative standard deviation; OIT—oxidation induction temperature; OIt—oxidation induction time; PLA_2_—phospholipase A_2_; QqQ—triple quadrupole; Q-TOF—quadrupole time-of-flight; RP-HPLC—reversed-phase high-performance liquid chromatography; SPE—solid-phase extraction; TLC—thin-layer chromatography; TNF-α—tumor necrosis factor α; TOF—time of flight; UPLC-MS—ultra performance liquid chromatography–mass spectrometry; UV–Vis—ultraviolet–visible spectroscopy.
